# Congenital Absence of the Left Main Coronary Artery in a 67-Year-Old Healthy Male: A Case Report and Mini Review

**DOI:** 10.7759/cureus.30563

**Published:** 2022-10-21

**Authors:** Amaka Sowemimo, Endurance O Evbayekha, Anita O Benson, Adebola G Adedeji, Okelue E Okobi

**Affiliations:** 1 General Medicine, University of Maiduguri Teaching Hospital, Maiduguri, NGA; 2 Internal Medicine, St. Luke's Hospital, St. Louis, USA; 3 Internal Medicine, Delta State University Teaching Hospital, Delta, NGA; 4 General Practice, Olabisi Onabanjo University, Ago Iwoye, NGA; 5 Family Medicine, Arizona State University, Tempe, USA; 6 Family Medicine, Lakeside Medical Center, Belle Glade, USA

**Keywords:** anatomy of anomalous coronary artery, collaterals, aberrant left coronary artery, anomalous origin of the left coronary artery from pulmonary artery, anomalous origin of the left coronary artery from pulmonary artery (alcapa)

## Abstract

Congenital absence of the left main coronary artery is a very rare entity. The literature surrounding this presentation describes it as a fairly common cause of sudden death especially in early life. Some schools of thought hypothesize that for the few cohorts who live into their adulthood with this anomaly, serious cardiovascular complications usually ensue.

We present a case of a generally healthy and asymptomatic 67-year-old gentleman with a history of diagnosed congenital absence of the left main coronary artery since age six without any active cardiovascular complaints at baseline.

## Introduction

Congenital absence of the coronary arteries has a low incidence and prevalence in society, with a 0.3-5.6% prevalence [1]. The incidence of a congenital absent left main coronary artery is between 0.02% to 0.07% in individuals undergoing coronary angiography [2]. These groups of anomalies may be benign or life-threatening.

Some studies suggest that this anomaly group is a leading cause of death through premature atherosclerosis in some young, otherwise healthy athletes [3]. The diagnosis may be done using transthoracic echocardiography, and in the situation where this is equivocal, other approaches, including transesophageal echocardiography, computed tomographic (CT) angiography and cardiac magnetic resonance imaging (CMRI) is useful [3].

## Case presentation

This is a 67-year-old gentleman referred to our facility for cardiovascular risk factor assessment. He was referred by his primary care physician (PCP) for an abnormal electrocardiogram (EKG) (inferior ST elevation, maybe ischemia vs. nonspecific changes). He denied substernal chest pressure, orthopnea or paroxysmal nocturnal dyspnoea (PND), or syncopal episodes. He described himself as being asymptomatic during his baseline activities. His past medical history is significant for anomalous origin of the left coronary artery from the pulmonary artery at age six. There is also a history of surgical intervention to graft the aberrant left coronary artery with the aorta, which was unsuccessful, leaving him with only a right coronary artery. He has a twin brother who is doing fine and denies any family history of heart disease. He has no known drug allergies. He is a nonsmoker, consumes moderate alcohol, and swims when the weather permits. 

On physical examination, he appeared to be in no obvious distress, lungs were clear to auscultation, blood pressure was within normal range, the precordial exam was essentially unremarkable, revealing normal S1 and S2, no murmurs or gallop, heart rate averaged 69 beats per minute, jugular venous pressure (JVP) was flat. EKGs mostly showed normal sinus rhythm with no acute changes. A bedside echocardiogram revealed an ejection fraction of 50-55% with no significant valvular lesions.

Stress tests

**Figure 1:**
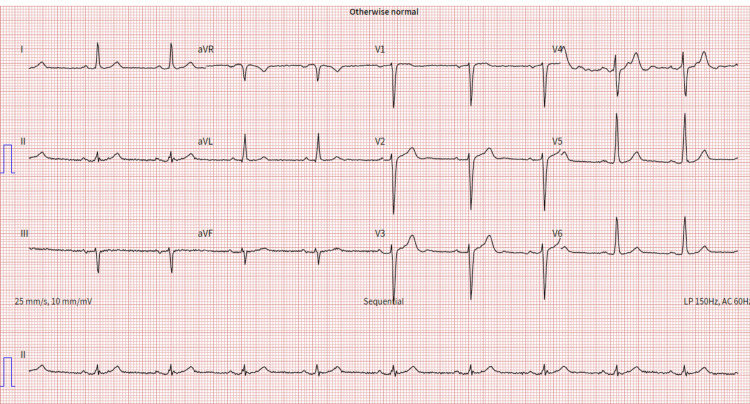
Electrocardiograph reading shows left axis deviation

**Figure 2:**
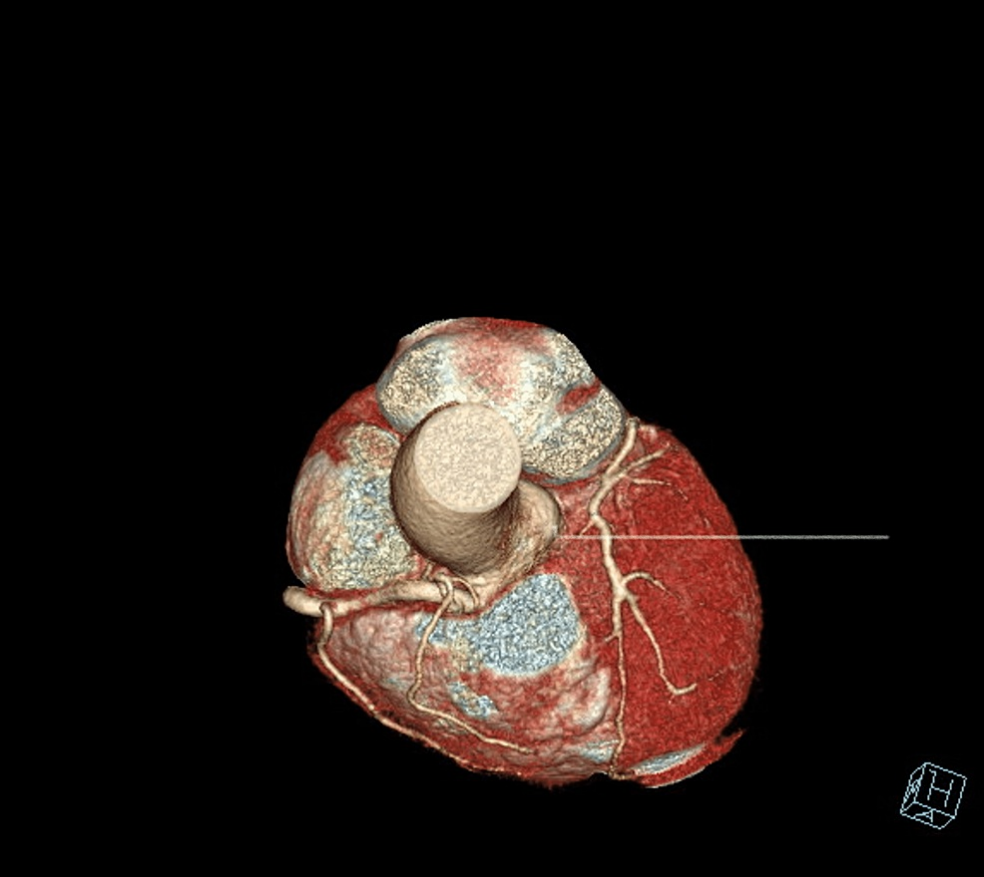
Single Coronary Artery Arising From The Right Coronary Sinus of Valsalva, no LMA Identified LMA= Left Main Artery

**Figure 3:**
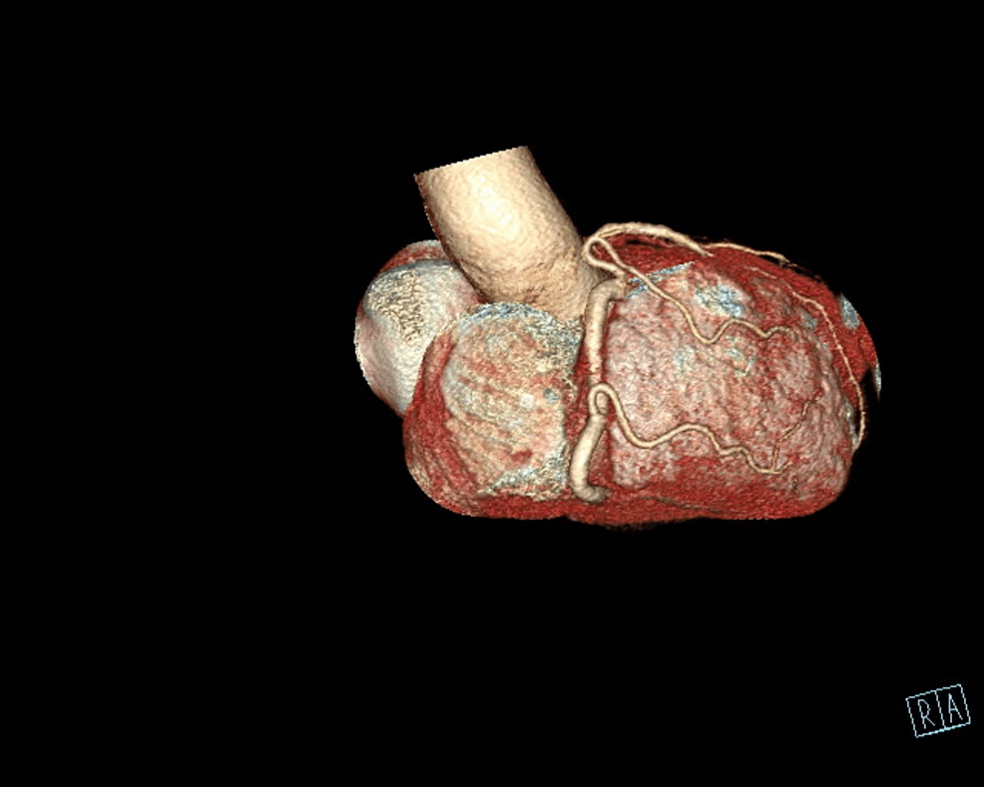
Posterior Descending Artery With Collateral Flow to the Left Side of the Heart LAD= Left Anterior Descending Artery

**Figure 4:**
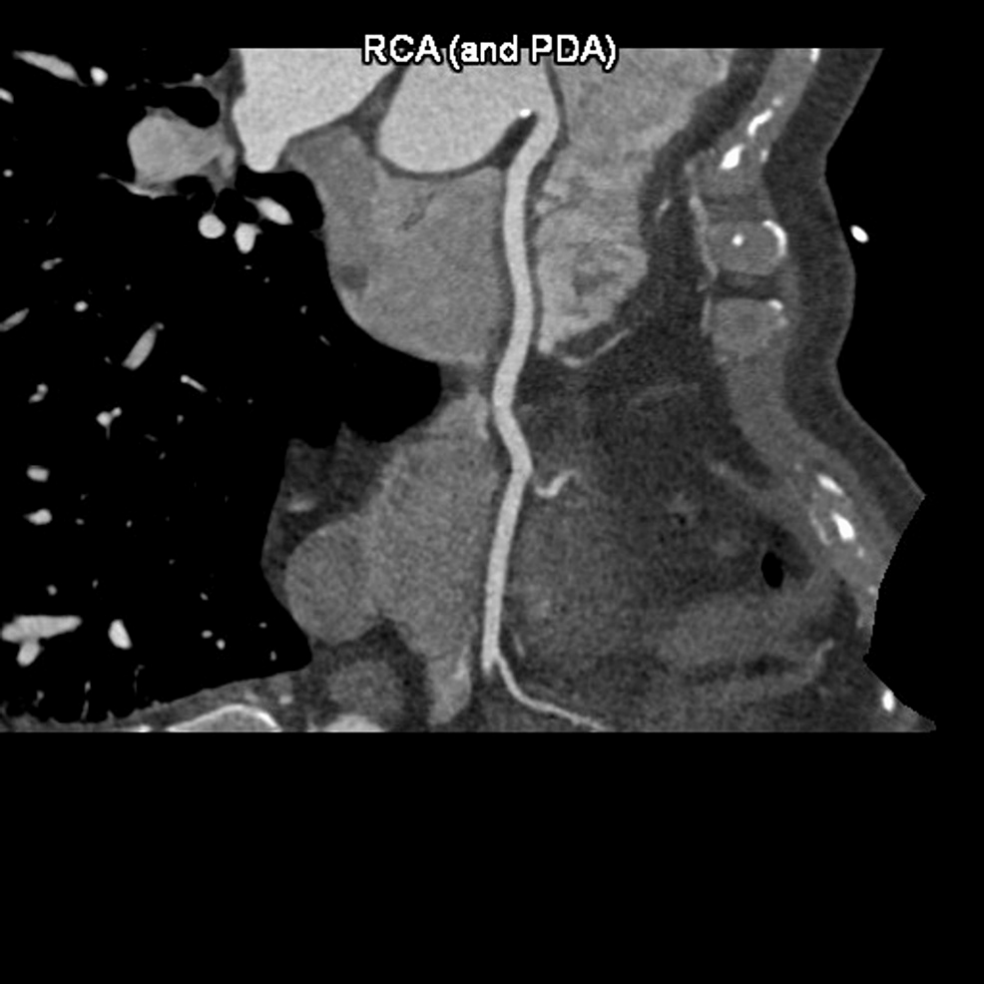
Right Coronary Artery to Posterior Descending Artery Anatomy RCA= Right Coronary Artery PDA= Posterior Descending Artery

The patient underwent an initial stress test and achieved 13 metabolic equivalents (Mets) of workload, which is good functional capacity, the hemodynamic response was normal, and there were no ischemic ST changes at the peak of exercise or recovery. A repeat stress test done one year later revealed some ischemic ST changes at the peak of physical exertion. Still, his resting EKG was normal except for mild left axis deviation, as seen in Figure 1 below. The ST changes on the stress test prompted further investigation with a coronary multi-slice CT angiogram. This revealed a single coronary artery that arises from the right coronary sinus of Valsalva, as seen in Figures 2, 3, 4. The right coronary artery (RCA), posterior descending artery (PDA), and posterolateral branch appeared to be patent. We suspected there might be a collateral flow from the posterolateral branch to the left circumflex and from the PDA to the left anterior descending artery (LAD). Collateral pathways were not well assessed, and the left circumflex and LAD were poorly assessed due to their small size. His only active cardiovascular medication was atorvastatin 40mg.

In summary, the patient is a 67-year-old male with a congenital anomaly of the left coronary artery with collaterals from the RCA who is managed for cardiovascular risk factors. He continued to be stable from a cardiac standpoint.

## Discussion

Embryological sequence of coronary artery formation and possible variants of similar anomalies

The process of coronary vessel formation involves an array of events that include de novo formation of blood vessels (vasculogenesis), formation of new capillaries (angiogenesis), and the transformation or remodeling of pre-existing arterioles into functional collateral arteries (arteriogenesis) [4]. Several small endothelial channels merge to form the coronary vessels [5,6]. The tips of these vessels advance towards the left and right aortic sinuses, penetrate the tunica media of the aorta, pierce the aortic endothelial lining, and establish continuity with the aortic lumen to form the right and left coronary arteries. While it is worthwhile to note that penetration of the aorta by the tubular network is complex and perfectly coordinated, only two major coronary arteries are formed, and variants and anomalies occur [4,6]. 

Coronary artery variants and anomalies

The two main coronary arteries are the right and left coronary arteries (LCA), with the LCA giving rise to the left circumflex and left anterior descending arteries. Anomalies of the coronary artery and variants remain the second most common cause of cardiac death in young competitive athletes, despite being uncommon [1,3]. The anomalous origin of a coronary artery from the contralateral sinus is the anomaly most frequently associated with sudden cardiac death. Other coronary artery anomalies, such as the anomalous origin of the left coronary artery from the pulmonary artery, as seen in the case under review, atresia of the left main stem, and coronary fistulae, have also been implicated in cases of sudden cardiac death [3,4,6]. Most patients are usually asymptomatic, with incidental discovery of the anomalies during coronary angiography or on autopsy following sudden cardiac death. Some symptoms, such as angina, syncope, heart failure, and myocardial infarction, may occur [3-5].

Anomalous origin of left main coronary artery from the pulmonary artery

Anomalous origin of the left main coronary artery from the pulmonary artery (ALCAPA) is also known as Bland-White-Garland syndrome and was described for the first time in 1956 [7]. ALCAPA is a very rare congenital coronary artery anomaly (0.008%), characterized by extensive collateral formation from the right coronary artery to areas usually supplied by the left coronary artery [4,7]. In the neonatal period, physiologic pulmonary hypertension preserves anterograde flow within the left coronary artery, and infants are usually asymptomatic. As pulmonary pressure drops, left-to-right shunting from the higher-pressure left coronary arterial system to the lower-pressure pulmonary arterial system occurs, and patients become symptomatic. Ninety percent of patients with this anomaly die during the first year of life, and very few survive into adulthood [7]. According to some authors, this could be explained by the inadequate development of inter-coronary collaterals. However, when these collaterals develop, as seen in the case under review, prognosis and overall outcome improve. 

Treatment guidelines and prognosis

Like many pathologies, the prognosis varies individually, depending on the extent of collateral formation. Unfortunately, most infants die within the first year [8]. Circulatory insufficiency is the cause of mortality in the majority. This insufficiency may be from left ventricular dysfunction, myocardial infarction, life-threatening arrhythmias, or mitral valve insufficiency [9]. 

Timely surgical intervention is potentially curative. Creating an aortopulmonary window and intrapulmonary tunnel, baffling the aorta to the ostium of the abnormal left coronary artery, is usually the most adopted surgical repair approach. It is called the Takeuchi procedure [7]. The Takeuchi procedure employs the anastomosis of the anomalous left coronary artery directly to the aorta. It was first described in the 1970s and still remains the current surgical approach [7]. 

Anomalous coronary artery evaluation and recommendations (AHA/ACC)

Computerized tomography angiography (CTA), cardiac magnetic resonance (CMR), and catheterization are great options for delineating the proximal course of the coronary artery and its relativity to surrounding structures. CTA is preferred because of its superior spatial and temporal resolution. However, CMR may also be adequate enough to delineate the relationship of the anomalous coronary artery to the pulmonary artery, aorta, and other structures. In the setting of concern for stenosis in the anomalous coronary artery or when hemodynamic evaluation of a shunt, coronary angiography by catheterization can be helpful [10].

Recommendations for anomalous coronary artery arising from the pulmonary artery

Surgical interventions may include direct reimplantation of the left coronary artery into the aorta with or without an interposition graft. Closure or ligation of the left coronary artery from the point of origin in the pulmonary artery with coronary artery bypass grafting may be a reasonable alternative. The left internal mammary artery can be used as a conduit to anastomose to the left anterior descending [10].

Surgery may involve reimplanting the right coronary artery into the aorta. An interposition graft may or may not be employed for this purpose. Closure or ligation of the right coronary artery from the pulmonary artery by coronary artery bypass grafting (CABG) is another option that can be performed. CABG's right internal mammary artery is usually anastomosed to the posterior descending coronary or right coronary arteries [10].

Surgical interventions such as reimplanting the right coronary artery directly into the aorta with or without an interposition graft may be reasonable for ischemic disease or ventricular dysfunction likely from the anomaly. Closure or ligation of the right coronary artery from the PA with a coronary artery bypass grafting using the right internal mammary artery for anastomosis to the posterior descending or right coronary artery [10]. 

Determinants of survivability and outcome

Determinants of survivability are directly related to the degree and caliber of the collaterals from the right main coronary artery. The right coronary circulation is usually large and supplies the left anterior descending and left circumflex arteries via one or more collateral vessels. Anterior collaterals may sometimes follow a malignant course, arising from the proximal right coronary artery or anterior coronary sinus and passing between the posterior aspect of the right ventricular outflow tract (RVOT) and the anterior aspect of the aortic root. This has been reported to confer additional risk of sudden death [11]. Another variant of the collateral vessels associated with sudden cardiac death is one that courses between the root of the aorta and the pulmonary trunk and is at risk of mechanical compression by these two arteries [12].

The presence of associated cardiac anomalies can also be a determinant of survivability. In the majority of the pediatric population with atresia of the left main coronary artery, the atresia coexists with supravalvular aortic stenosis, ventricular septal defect, pulmonary stenosis, prolapse of the mitral valve or patent ductus arteriosus, this gives a poor prognosis compared to an isolated defect seen in the older population [13]. Early access to cardiovascular imaging and interventions also alters the course of the disease. It influences the survivability of the condition even in cases where it is an incidental finding and the patient is essentially in good cardiovascular health.

A few common outcomes are expected with the absence of the left main coronary artery, especially in older patients. The first is sudden cardiac death which has been reported in increasing frequency by existing literature [11,14]. One study reported intermittent chest discomfort and an inconclusive exercise treadmill test [15]. Existing literature has also reported left ventricular dysfunction and subsequent heart failure [13]. The patient can also be asymptomatic, as seen in our case. Again, the caliber and location of the collaterals play a great role in the long-term prognosis of these patients and their non-structural cardiovascular risk.

## Conclusions

The congenital absent left main coronary artery is a rare entity, and some schools of thought have postulated that it presents a variety of cardiovascular complications that may be life-limiting. Theoretically, unfortunately, those who have this condition are expected to manifest symptoms in early life. We have presented a case of an otherwise healthy 67-year-old gentleman with a confirmed diagnosis of congenital absence of the left coronary artery since age six. This may give clinicians an alternative insight into the prognosis of this understudied population.
